# Supplementary material for: Splice-Junction-Based Mapping of Alternative Isoforms in the Human Proteome
Source: Cell Rep. Author manuscript; Available in PMC 2020 Jan 15. (PMC6961840; doi:10.1016/j.celrep.2019.11.026)
Supplement: 3 [file NIHMS1546469-supplement-3.zip › DF2/PXD009737/TestisGluC-206-Q14114-YPARVALSLE.pdf]

A

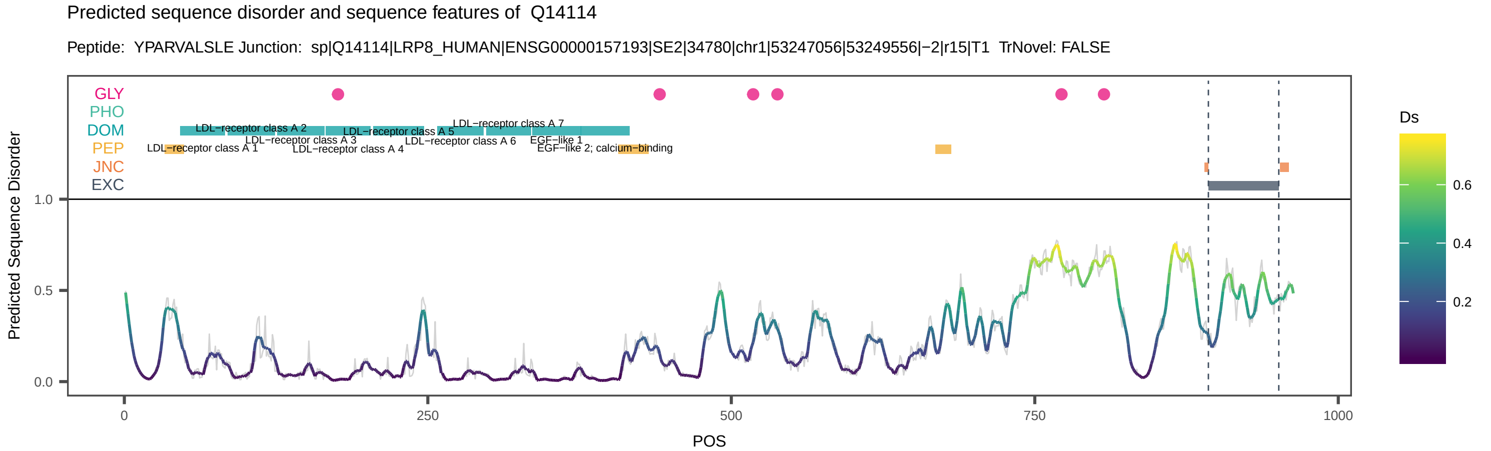

Predicted sequence disorder and sequence features of Q14114
Peptide: YPARVALSLE Junction: sp|Q14114|LRP8_HUMAN|ENSG00000157193|SE2|34780|chr1|53247056|53249556|−2|r15|T1 TrNovel: FALSE
GLY
PHO
DOM
PEP
JNC
EXC
LDL−receptor class A 1
LDL−receptor class A 2
LDL−receptor class A 3
LDL−receptor class A 4
LDL−receptor class A 5
LDL−receptor class A 6
LDL−receptor class A 7
EGF−like 1
EGF−like 2; calcium−binding
Predicted Sequence Disorder
1.0
0.5
0.0
0
250
500
750
1000
POS
Ds
0.6
0.4
0.2


B

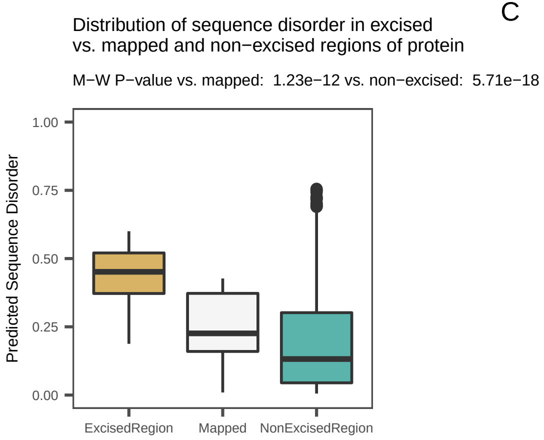

Distribution of sequence disorder in excised
vs. mapped and non−excised regions of protein
M−W P−value vs. mapped: 1.23e−12 vs. non−excised: 5.71e−18
Predicted Sequence Disorder
1.00
0.75
0.50
0.25
0.00
ExcisedRegion
Mapped
NonExcisedRegion


C
